# Parkinson’s-adapted cognitive stimulation therapy: feasibility and acceptability in Lewy body spectrum disorders

**DOI:** 10.1007/s00415-019-09329-6

**Published:** 2019-06-04

**Authors:** Sheree A. McCormick, Sabina Vatter, Lesley-Anne Carter, Sarah J. Smith, Vasiliki Orgeta, Ellen Poliakoff, Monty A. Silverdale, Jason Raw, David J. Ahearn, Christine Taylor, Joanne Rodda, Tarek Abdel-Ghany, Benjamin Kwapong, Iracema Leroi

**Affiliations:** 1 Division of Neuroscience and Experimental Psychology, University of Manchester, Jean McFarlane Building, Oxford Road, Manchester, M13 9PL UK; 2 Division of Population Health, Health Services Research & Primary Care, University of Manchester, Manchester, UK; 3 School of Health and Community Studies, Leeds Beckett University, Leeds, UK; 4 Division of Psychiatry, University College London, London, UK; 5 Salford Royal NHS Foundation Trust, Salford, UK; 6 Pennine Acute Hospitals NHS Trust, Oldham, UK; 7 Manchester University NHS Foundation Trust, Manchester, UK; 8 Derbyshire Healthcare NHS Foundation Trust, Derby, UK; 9 North East London NHS Foundation Trust, London, UK; 10 North West Boroughs Healthcare NHS Foundation Trust, Winwick, UK; 11 Greater Manchester Mental Health NHS Foundation Trust, Manchester, UK; 12 Global Brain Health Institute, Trinity College Institute of Neuroscience, Trinity College Dublin, Lloyd Building, Dublin 2, Ireland

**Keywords:** Parkinson’s disease dementia, Feasibility, Psychosocial therapy, Cognition, Quality of life

## Abstract

**Background:**

Drug-based therapeutic approaches for Parkinson’s disease dementia (PDD) and dementia with Lewy bodies (DLB) are moderately effective and not always tolerated. Tailoring psychosocial approaches in PDD and DLB may offer additional support and improve outcomes. We adapted home-based, care partner-delivered Cognitive Stimulation Therapy (CST) for individuals with PDD or DLB and their care partners (CST-PD).

**Objectives:**

To evaluate the feasibility, acceptability, and tolerability of CST-PD.

**Methods:**

This randomised controlled trial used mixed methods, including a process evaluation. People with PDD, DLB or mild cognitive impairment in PD (PD-MCI) and their care partners were randomised to 12 weeks of treatment as usual (TAU) or CST-PD. Outcomes were feasibility of the study conduct (i.e., recruitment, retention rate) and acceptability and tolerability of the intervention. Measures included rating scales, researcher field notes, therapy diaries, and exploratory clinical and care partner efficacy measures.

**Results:**

The recruitment target was met with 76 consenting participant-dyads. Retention in both arms was high at over 70%. More than 90% of dyads undertook discrete sessions greater than 20 min duration, but the average number of sessions completed was lower than the recommended dose. Acceptability ratings (i.e., interest, motivation and sense of achievement) of the intervention were high. Participants reported no serious adverse events related to the intervention.

**Conclusions:**

The field of psychosocial interventions for PDD and DLB is newly emerging, and we demonstrated that this type of intervention is acceptable and well tolerated. Evaluating its clinical effectiveness in a full-scale randomized controlled clinical trial is warranted.

**Trial registration number:**

The trial is a psychosocial intervention with an allocated ISRCTN number 11455062.

**Electronic supplementary material:**

The online version of this article (10.1007/s00415-019-09329-6) contains supplementary material, which is available to authorized users.

## Introduction

Each year about 11% of people with Parkinson’s disease (PD) develop dementia (PDD), which is likely to triple in prevalence by 2060 [1, 2]. The prevalence of dementia with Lewy bodies (DLB), which encompasses around 5% of dementias overall, is also increasing [3]. Treatment strategies for PDD and DLB have mostly focussed on medication-based interventions, such as cholinesterase inhibitors or memantine, with effectiveness of these treatments being modest at best. There is a need to find other management strategies specifically adapted for PDD, DLB and related cognitive impairment in PD (termed ‘Parkinson’s-related dementia’; PRD).

Cognitive Stimulation Therapy (CST) and cognitive rehabilitation therapy can enhance cognitive function, quality of life and care partner outcomes in people with non-Parkinson’s-related dementias [4, 5] but studies in PDD and DLB are limited [6–8]. We, therefore, designed the INVEST (INdiVidualised cognitivE Stimulation Therapy) programme to develop and evaluate a personalised psychosocial intervention specifically for people with PRD. The first phase of INVEST involved adapting the home-based version of CST known as individualised CST (iCST; [4]), for people with PRD and their care partners [9]. Here, in the second phase, we evaluated the newly adapted therapy, CST-PD, to inform a subsequent randomised controlled trial (RCT) of effectiveness. In accordance with the UK’s Medical Research Council’s guidelines for the development of complex interventions, [10] our objectives were to:

(i) conduct an evaluation of the operational aspects of the study including aspects of recruitment, retention, data collection and study design;
(ii) assess the acceptability and tolerability of the intervention;
(iii) evaluate intervention fidelity, i.e., whether the intervention could be delivered, received and enacted as intended [11];
(iv) explore a possible signal of efficacy.

The first three objectives are reported here, whereas the exploratory pilot trial of efficacy (objective iv) is reported elsewhere [12].

### Materials and methods of the trial

**Fig. 1:**
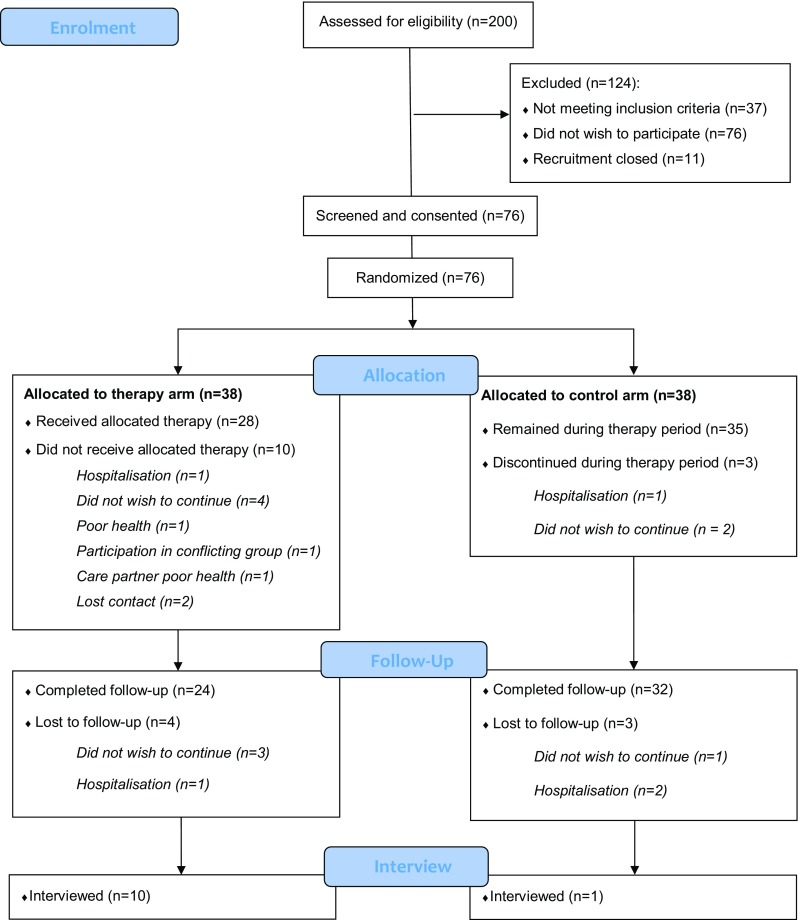
CONSORT 2010 flow diagram

The full study protocol has been described previously [13]. Participant-dyads were recruited in the UK from four sites in Greater Manchester (primary site), and three sites in London, Derby, and Warrington. A CONSORT diagram [14] outlines the participant flow through the study, Fig. 1.

#### Eligibility

Participants with mild cognitive impairment in PD (PD-MCI), PDD or DLB were eligible if they were aged 18 years or older, had a diagnosis based on standard clinical diagnostic criteria [15–17] determined by the referring PD specialist, were living at home, and were on stable medication for at least 4 weeks prior to study entry. Care partners were eligible if they were 18 years or older. Capability to participate was based on clinical impression during screening and informed by scores obtained on the Unified Parkinson’s Disease Rating Scale- motor examination [UPDRS-III; 18], the Schwab and England Activities of Daily Living Scale [SE-ADL; 19] and the Montreal Cognitive Assessment [MoCA; 20]. Participant-dyads were excluded if they could not understand conversational English, were non-literate, had a severe physical or psychiatric illness that precluded participation, or were taking part in another psychosocial intervention study.

#### Study design, randomisation and ethics

This was a single-blind parallel arm, exploratory pilot trial with an embedded feasibility study and process evaluation. An accredited clinical trials’ unit undertook blocked randomisation to CST-PD, or treatment as usual (TAU), on a 1:1 participant-dyad level, without stratification. All participants provided written consent, either voluntarily or via a personal consultee declaration. Demographic data and descriptive clinical measures were collected from participant-dyads during screening. Outcome measures (see Supplementary Table 1: Outcome measure descriptions for people with Parkinson’s-related dementia and care partners) were administered by assessors blind to treatment allocation. Assessors were asked to report instances of un-blinding to the study team. The study received favourable ethical opinion from the Yorkshire and The Humber–Bradford Leeds Research Ethics Committee (reference: 15/YH/0531).

#### Study interventions

Participants in the experimental arm were asked to complete 12 weeks (including a 2-week familiarization lead-in) of CST-PD, delivered at home by the care partner. The therapy involved participating in 30 min of cognitively stimulating activities, two to three times per week. The activities varied in theme and complexity and could be tailored to suit individual needs (see Supplementary Table 2: Themes and topics from the CST-PD manual). The TAU services, offered by the National Health Service in England, were available to those in the experimental arm. At the end of the intervention period, participant-dyads allocated to TAU were offered the opportunity to receive the intervention without data being collected.

#### Assessment battery

Clinical and care partner measures of potential efficacy, including measures of cognition, behavior, well-being and quality of life (Supplementary Table 1 and described in detail elsewhere [13]), were administered at baseline and immediately after the intervention period.

### Feasibility, acceptability, and tolerability evaluation framework

To evaluate INVEST we used a modified version of the ACCEPT [21] checklist based on Thabane et al.’s guidelines for reporting feasibility trials [22]. Our modified checklist provided a systematic basis for making decisions about whether or not to accept components of our study protocol ‘as is’ or whether to modify it, and in what way. We applied a series of operationalized definitions of feasibility, acceptability and tolerability, with a priori criteria, to arrive at a conclusion of ‘accept’, ‘modify’, or ‘reject’ (Table 2). These criteria were operationalized through a combination of pragmatic clinical judgements regarding acceptability in a population with PRD, as well as previous studies of nonpharmacological interventions for dementia and chronic mental illness [4, 30]. In particular, the ‘parent’ study of INVEST, a full scale RCT of iCST in people with dementia not related to Parkinson’s [4], provided valuable insight into appropriate interpretations of acceptability thresholds for the different aspects of the study. The ACCEPT framework was originally designed as a flexible approach that can be applied to pragmatic clinical trials for ‘real life’ populations regardless of specific diagnosis [2]. Thus, its application to the current study has been appropriate and useful.

#### Feasibility of the study procedures

‘Feasibility’ was operationalised through the question, ‘can it work?’ [23]. Parameters included: eligibility, recruitment and retention rates, overall trial design (the degree to which the protocol balanced scientific and practical considerations), willingness to be randomised, blinding procedures and data collection (i.e., timing, quality, acceptability).

#### Acceptability of the intervention

This was defined as the extent to which the participant-dyads considered the intervention ‘appropriate’ [24]. We assessed care partner’s perceptions of their partner’s interest, motivation and sense of achievement following each therapy session. These aspects were rated in the therapy diary on a Likert-scale, consisting of the following grades: ‘1’ = strongly disagree; ‘2’ = disagree; ‘3’ = neutral; ‘4’ = agree; ‘5’ = strongly agree (see Supplementary Table 3: Companion’s CST-PD diary excerpt).

#### Tolerability of the intervention

Tolerability was defined as the ability to endure the intervention [25] and was captured by the number of serious adverse events (SAEs) related to either member of the participant-dyad feeling burdened or frustrated.

#### Intervention fidelity (delivery, receipt and enactment)

Intervention delivery was operationalised through two questions, ‘can researchers deliver therapy training to care partners?’ and ‘can care partners deliver the therapy to their partners?’ Similarly, receipt was operationalised as, ‘did care partners receive the therapy training as intended?’ and ‘did participants with PRD receive CST-PD as intended?’ To evaluate enactment we asked, ‘did participant-dyads complete the recommended amount of therapy during the intervention period?’ Measures included a training checklist, a training evaluation form, a therapy skills self-assessment, and diary entries (acceptability ratings and free-text entries).

### Qualitative investigation

We conducted semi-structured interviews at the end of the intervention with a purposive sample of 11 participant-dyads who undertook the therapy to gather evidence to support or refute our conclusions regarding the categorisations of ‘accept’, ‘modify’ or ‘reject’. This sample size is considered sufficient to capture all emerging themes, as data saturation (rather than ‘theoretical saturation’) was likely to have been reached. We followed guidance suggesting that if participants are relatively homogeneous (e.g., as in our case, in which participants all fell within a single diagnostic spectrum and had a close age-range), then a purposive sample size approaching 12 will make it possible to ‘render a fairly exhaustive data set’ [26].

### Sample size

We applied guidance on sample sizes for exploratory work [27, 28] and took a conservative approach, estimating the standardised effect size on cognition of the parent form of the intervention to be 0.4, and utilizing a one-sided test and a less stringent significance level of 0.2 to avoid missing a promising effect. Thus, assuming 80% power and a correlation coefficient of 0.5 between baseline and endpoint on cognitive outcomes, the required sample size, including allowance for attrition, was 38 dyads per group at enrolment [13].

### Data analysis

Quantitative data were analysed as descriptive statistics, qualitative data were analysed using thematic analysis [29]. Theme identification and initial coding were performed by authors SAM, SV and BK. The coding framework was further developed and themes defined through an iterative process by the INVEST investigators (SAM, SV, SJS and IL). To reach consensus, alternative meanings and interpretations were discussed in the context of all transcripts.

## Results

### Feasibility of the study procedures

Findings from all data sources regarding the feasibility aspects of the study procedures are outlined in the modified ATTEND checklist, Table 1.

**Table 1:** Modified ACCEPT checklist for feasibility, acceptability and tolerability of trial design, study procedures and intervention

Component being evaluated and operationalized definition	A priori standard for acceptance (A), modification (M) or rejection (R)	Data source (how and when)	Descriptive outcome	Conclusion Accept/modify/reject
Feasibility of the trial design and study procedures				
Overall trial design To assess adherence to trial procedures	Feasibility will be demonstrated if the reported protocol deviations are not unethical, do not compromise data integrity and can be controlled in a future trial	Participant flow log^a^	The majority of protocol deviations were related to delays in scheduling the home visits. The delays extended the trial duration for some participant-dyads Control = 15 weeks, 5 days Experimental = 18 weeks, 1 day	Modify
Eligibility rate To estimate the number of eligible participant-dyads entering the study	A: >15%^A^ M: ≥10% R: <10%	Participant flow log^a^	200 participants were referred to the study, of these 76 entered the study. Thus eligibility rate was 38%	Accept
Recruitment rate To estimate the number of participant-dyads who can be recruited per site per month	A: > 2 dyads per month over 6 month period^A^ M: ≥ 1 dyad per month over 6 month period R: < 1 dyad for two consecutive months	Participant flow log^a^	1 dyad per month (actual – 0.9 dyads per site per month over 6 month period)	Modify
Randomisation To estimate the number of dyads accepting recruitment allocation	A: > 95% acceptance^A^ M: ≥ 90% R: < 90%	Participant flow log^a^	All participant-dyads accepted their allocated arm	Accept
Retention rate To estimate the number of dyads lost to follow up after entering the trial	A: > 70%^A^ M: ≥ 60% R: < 60%	Participant flow log^a^	The retention rate was 73.7% (20 out of 76) participant-dyads were not followed up. Of the 20 participant-dyads not followed up, 70% (14 participant-dyads) were in the experimental arm and 30% (6 participant-dyads in the control arm	Accept
Blinding procedures To estimate the percentage of ‘un-blinding’ incidents reported by assessors	A: < 10% incidents of un-blinding reported M: ≤ 15% incidents of un-blinding reported R: > 15% incidents of un-blinding reported	Field notes^a^	There were no reports of un-blinding at any of the 7 sites	Accept
Data collection To assess the feasibility of outcomes assessments by participant-dyads and assessors	Feasibility demonstrated if outcome measures assessments can be administered within 120 min ± 10% (as per ethical approval) and level of complete data > 80%	Assessor record form^c^ Summary statistics^h^	Average time to complete assessment battery: 122.4 min (SD = 23.0 min, range 76–150 min) Missing outcome scale data for trial completers were minimal and met the a priori threshold for acceptability, with all scales having over 83% complete data	Accept
Usability of participant diaries To assess the feasibility of using a field diary to record therapy experiences	A: Feasibility demonstrated if the diaries from > 95% of therapy completers are returned to the study team, > 40% completed M: Partial feasibility demonstrated if ≥ 85% of all diaries are returned to the study team, > 40% completed R: Feasibility not demonstrated if < 85% of diaries are not returned to the study team, and/or < 40% completed	Therapy diary^e^	Diaries were returned for 91.7% of completers (22/24 participant-dyads) in the experimental arm. Participant-dyads recorded diary entries for all completed therapy sessions	Accept
Intervention fidelity				
Delivery To assess the feasibility of researchers delivering the therapy training as per the protocol guidelines To assess the feasibility of care partners delivering the therapy to participants with Parkinson’s-related dementia	A: Fidelity demonstrated if > 95% of assessors complete the activities on the protocol check-list M: Partial fidelity demonstrated if ≥ 75% of assessors complete the activities on the protocol check-list R: Fidelity not demonstrated if < 75% of assessors do not complete the activities of the protocol check-list Feasibility demonstrated if the majority of care partners report that they delivered the therapy in accordance with the key therapy principles	Researcher check-list^d^ Semi-structured interviews^g^	77.8% of assessors completed the check-list at three sampled sites. From the check-list, 82.1% of assessors delivered the therapy training as intended, the remainder could not complete a role-play exercise with the study partner due to lack of time Some care partners reported difficulty in delivering the therapy to their partner and rarely revisiting the key therapy principles outlined in the therapy manual	Modify Modify
Receipt To assess the feasibility of care partners receiving the therapy training in the manner intended To assess the feasibility of participants with Parkinson’s-related dementia receiving CST-PD as intended (see Acceptability components below)	A: Fidelity demonstrated if > 95% of care partners rate their skills (post-training) > 3 M: Partial fidelity demonstrated if ≥ 75% of participants rate their skills (post-training) as > 3 R: Fidelity not demonstrated if < 75% of participants rate their skills (post-training) as > 3 See acceptability component below as a proxy measure of receipt of CST-PD by participants with Parkinson’s-related dementia	Therapy skills self-assessment^d^	95.6% of care partners rated their skills (post-training) as ≥ 3	Accept
Enactment To estimate the number of therapy sessions completed	A: Fidelity demonstrated if > 95% of participants complete 11 sessions of 20 min or longer M: Partial feasibility demonstrated if ≥ 75% of participants complete 11 sessions of 20 min or longer R: Fidelity not demonstrated if < 75% of participants do not complete 11 sessions of 20 min or longer	Therapy diary^e^	64% of participants completed 11 sessions or more over the 10-week intervention period. 92% of participant-dyads completed sessions of greater than 20 min. Additional strategies will be required to boost the number of sessions completed	Modify
Acceptability, tolerability and feasibility (from the participant-dyad’s perspective) of the intervention				
Acceptability To assess study care partners’ perceptions of the extent to which (in each session) their partner was: interested, motivated and gained a sense of achievement	A: Acceptability demonstrated if > 95% of care partners rated each element 3 or more M: Partial acceptability demonstrated if ≥ 75% of care partners rated each element 3 or more R: Acceptability not demonstrated if < 75% of care partners do not rate each element 3 more	Therapy diary^e^	Partner was interested: 100% rated > 3 (64% rated > 4) Partner was motivated: 100% rated > 3 (60% rated it > 4) Partner gained a sense of achievement: 96% >3 (40% greater than 4)	Accept
Tolerability To estimate the number of serious adverse events related to the trial	A: Feasibility demonstrated if any serious adverse events reported are not related to tolerability of the intervention^A^	Participant flow^a^ log, field notes^a^	No serious adverse events reported to the study team were related to the trial	Accept
Feasibility To assess the feasibility of delivering the intervention in a way that is acceptable to people with Parkinson’s-related dementia and their care partners	Feasibility demonstrated if the majority of participant-dyads indicate that the therapy is acceptable, usable and tolerable	Semi-structured interviews with 11 participant-dyads who undertook the therapy^g^	Most participants found the CST-PD enjoyable, usable, stimulating and versatile, confirming the acceptability and tolerability of the therapy (see Table 3). However, a number of barriers were identified, such as lack of time to deliver the intervention, poor communication skills and disease-related aspects preventing the dyad from engaging in therapy sessions	Modify

The timings of the assessment for each component were as follows: ^a^ongoing throughout study, ^b^informed consent, ^c^baseline assessments, ^d^therapy training, ^e^10-week intervention period, ^f^follow up assessments, ^g^after 10-week intervention, ^h^study end

^A^Based on findings in comparable studies investigating psychosocial interventions in dementia [4, 30]

### Recruitment/randomisation

The recruitment rate in the first 10 months was 0.8 dyads per site per month, which was lower than expected, and likely influenced by seasonal variation and holidays. Thus, three additional sites were added to enable us to recruit to target at a rate of 0.9 dyads per site per month, approaching the ‘modify’ threshold of 1–2 dyads per site. Of the 200 participant-dyads referred to the study team, 37 were ineligible, 11 were referred after recruitment had ended and 76 were unwilling to participate. Reasons for ineligibility included: no diagnosis of PD or cognitive impairment in PD; distance from the study centre; being admitted to a care home; and having a medical or psychiatric condition too severe to enable study participation. Reasons for non-participation by eligible people included: health issues; worsening dementia and apathy in the participant; care partner burden; administrative errors leading to failure to contact the dyad; and unwillingness to be randomised.

All participants (*n* = 76) had the capacity to consent to participation at the start of the trial. During the trial, four participants lost the capacity to consent and a consultee declaration form for continuation was completed by their care partners. Of the 76 participant-dyads consented, 38 were randomised to the CST-PD and 38 to TAU, none withdrew due to group allocation. These data suggest participant-dyads were willing to be randomised.

Of the participants with PRD, 19.8% (*n* = 15) had a diagnosis of PD-MCI, 52.6% (*n* = 40) had PDD, and 27.6% (*n* = 21) had DLB. Of this group, 79% (*n* = 60) were male, 93.4% were white (*n* = 71) with a mean age of 74.5 years (SD = 6.74). Of the care partners, 84.2% (*n* = 65) were living with their study partner, 11% (*n* = 8) were male; 77.6% (*n* = 59) were spouses or partners, 17.1% (*n* = 13) were relatives and the remainder 5.3% (*n* = 4) included a live-in carer, a live-in divorcee, a friend and a grandchild. Baseline demographics, per arm, are shown in Table 2. There was some variability, notably the participant-dyads in CST-PD had a higher level of schooling and a slightly higher proportion of people with PDD. The average number of years members of dyads were known to each other was slightly lower in CST-PD. This case mix imbalance suggests that randomisation was not fully effective.

**Table 2:** Baseline demographic and clinical variables in the experimental (CST-PD) and control (TAU) arms

Categorical variables	People with Parkinson’s-related dementia (*n* = 38)				Care partners (*n* = 38)			
	Control		Intervention		Control		Intervention	
	*n*	%	*n*	%	*n*	%	*n*	%
Gender								
Female	8	12.05	8	21.05	35	92.11	33	86.84
Male	30	78.95	30	78.95	3	7.89	5	13.16
Ethnicity								
White	35	92.11	36	94.74	35	92.11	35	92.11
Non-White	2	5.26	2	5.26	2	5.26	3	7.89
Did not specify	1	2.63	0	0.00	1	2.36	0	0.00
Education level								
Up to 18 year old schooling	22	57.89	18	47.37	20	52.63	17	44.74
Further education and higher	16	42.11	20	52.63	18	47.37	21	55.26
Marital status								
Single	6	15.79	6	15.79	7	18.42	6	15.79
Married/partnership	32	84.21	32	84.21	31	81.58	32	84.21
Living status								
Alone	5	13.16	1	2.63	2	5.26	0	0.00
With others	33	86.84	37	97.37	36	94.74	38	100.00
Diagnosis								
PD-MCI	8	21.05	7	18.42				
PDD	18	47.37	22	57.89				
DLB	12	31.58	9	23.68				
Dyad relationship								
Spouse/partner					28	73.68	31	81.58
Son/daughter					9	23.68	4	10.53
Other					1	2.63	3	7.89
Caregiving weekly hours (up to an average of)								
1 h per day					10	26.32	5	13.16
8 h per day					9	23.68	13	34.21
24 h a day					19	50.00	20	52.63

Continuous variables	*n*	Median; IQR [range]	*n*	Median; IQR [range]	*n*	Median; IQR [range]	*n*	Median; IQR [range]
Age	38	75; 72–81 [61–90]	38	74.50; 68–77 [55–84]	38	68.50; 59–72 [43–85]	38	67; 59–71 [21–88]
Dyad known years					29	50; 43–56 [3–68]	34	46; 30–52 [0.5–70]
Caregiving years					38	2.50; 1–6 [0–15]	38	3.25; 1.5–8 [0–20]
Montreal Cognitive Assessment (MoCA)	35	19; 15–22 [7–24]	36	17.5; 15–21.5 [8–30]				
Schwab-England score	37	60; 35–80 [10–100]	37	60; 30–70 [10–90]				
UPDRS motor score	38	34; 17.50–40.25 [9–69]	37	24; 18–38 [8–58]				
Duration of clinical symptoms, years	38	5.5; 2–10 [0–33]	38	4; 2–10.50 [0.5–24]				

### Eligibility criteria

Of the 200 participant-dyads referred to the study (see Fig. 1), 76 dyads were contacted and screened for eligibility. Of these, all met eligibility criteria and were subsequently randomised. This resulted in an eligibility rate of 36%, meeting the ‘accept’ threshold.

### Retention

The retention rate was 73.7% and meets the ‘accept’ threshold; 56 of 76 consented participant-dyads completed the trial. Of those who were not followed up, 70% (14 of 20) were in CST-PD and 30% (6 out of 20) were in TAU. Out of the total consented in each inclusion group, 25% (10 of 40) who were not followed up had PDD, 20% (3 of 15) had PD-MCI, and 14% (3 of 21) had DLB. Deteriorating health was the main reason for withdrawal across arms and inclusion groups. Three dyads withdrew from CST-PD as they found it difficult to discuss topics spontaneously or did not like reminiscing.

### Study design

We estimated that participant-dyads would remain in the trial for 15 weeks ± 2 weeks. This was achieved in TAU (15 weeks, 5 days), but not in CST-PD (18 weeks, 1 day). In CST-PD, the additional home-visit for therapy training introduced unforeseen delays mainly due to unavailability of participant-dyads.

### Blinding

No instances of un-blinding were reported to the study team across sites.

### Feasibility of outcome measures

The majority of outcome measures were feasible to administer; however, Goal Attainment Scaling (GAS [30]) was removed early in the trial as assessors reported that it was difficult to determine person-centred and measurable goals in the timeframe allocated. An early audit of the Addenbrooke’s Cognitive Examination-III (ACE-III [31]) at the primary site identified inconsistencies in scoring between assessors. To improve accuracy and maintain consistency, all assessors received additional training and a scoring checklist. Missing outcome scale data for trial completers were minimal and met the a priori threshold for acceptability, with all scales having over 83% complete data. Subsequent analyses of the data revealed no differential missingness conditional on the baseline factors [13].

The therapy diary was feasible in terms of recording participant-dyads experiences; 91.7% of diaries were returned by study completers who undertook the therapy, entries were recorded for all completed therapy sessions. Qualitative investigation supported these findings, for example:

“It would have been useful to have had more room for feedback on the diary, yes, um, ‘cause I’d sort of squashed quite a lot in there.” [Care partner, CS17].

### Acceptability of the intervention

The intervention was acceptable, based on the care partners’ perceptions of the extent to which (in each session) their partner was interested, motivated, and gained a sense of achievement. All a priori targets were exceeded; at least 75% of care partners rated the aspects within the target range (see Table 1). Qualitative findings support the diary ratings, for example:

“[name] chose an article he had read in an aeroplane magazine and showed much more interest and enthusiasm in. It is a subject dear to his heart.” [Care partner, CS17].

### Tolerability of the intervention

Of the 9 SAEs reported, 5 were from CST-PD and 4 from TAU. None of the SAEs reported were due to the trial, suggesting that the intervention was well tolerated.

### Intervention for TAU completers

All participant-dyads who completed their TAU arm (*n* = 32 dyads) were offered the intervention and 50% of dyads received the intervention. Data from these participants, however, was not collected due to ethical constraints.

### Intervention fidelity

#### Delivery and receipt of therapy training

The training protocol checklist revealed that 82.1% of researchers delivered CST-PD training as intended, the remainder completed all but the role-play exercise due to lack of time. The a priori target of CST-PD training being received as intended by the care partner was exceeded; after therapy training 95.6% of care partners (*n* = 23) rated their CST-PD skills within the ‘accept’ threshold. Furthermore, the training evaluation form, completed anonymously by care partners (*n* = 23), revealed that 95.4% found CST-PD training acceptable and 76.2% felt that they had received adequate instruction about how to deliver CST-PD.

Concerning the delivery of CST-PD by the care partner and receipt by the person with PRD, qualitative investigation revealed that some care partners struggled with personalising the activities, and/or coping with their partner’s disease symptoms. For example:

- *[name] took more interest in the subject today and it was less like ‘pulling teeth’. [...] He seemed to enjoy the whole session more today. [Care partner, CS17].*
- *Have we done it right? I felt sometimes that maybe I was the problem. Maybe I wasn’t approaching this right and I wasn’t getting the best out of [him]. [Care partner, CS4].*

It is possible that the challenges faced by some care partners may have influenced the receipt of CST-PD by the person with PRD, for example:

- *You’d come to the question and you’d say, “Well, I’ve already said that”, so what else can I say? [Participant with PRD, CS5].*

That said, the acceptability ratings for interest, motivation and sense of achievement (reported above) were high, suggesting that people with PRD received the therapy in the manner intended.

#### Enactment

During the intervention period, 64% of participants completed 11 or more sessions, with the average number of sessions being 1.76 (SD = 0.72) per week. The majority (92%) of participant-dyads completed sessions of longer than 20 min. Thus, the *‘modify’* target threshold for enactment was met. The most frequently cited barriers to enactment included disease symptoms and availability. For example:

- “A barrier for you, dad, would have been two things. One that you wanted to do the session but I wasn’t here to do one, so lack of availability of me, and second your health because your health varies so much day to day.” [Care partner, CS9].

### Qualitative findings

Eleven participant-dyads, who had completed the 10-week CST-PD protocol, were interviewed. Of these 11 dyads, one dyad completed the CST-PD intervention following their 10-week TAU participation. Of the people with PRD, six males had a diagnosis of PD-MCI, four had a diagnosis of PDD, and one had a diagnosis of DLB. The median age of participants with PRD was 72.0 (interquartile range [IQR] = 68.5–77.0) years, median MoCA score of 21.0 (IQR = 17.0–24.5), median H&Y stage of 3.0 (IQR = 1.8–3.5), and median years since PD diagnosis of 8.0 (IQR = 3.0–11.0) years. With respect to care partners, the sample comprised 11 females with a median age of 67.0 (IQR = 65.0–70.5) years, of whom nine were spouses/life partners and two were adult children. The semi-structured interviews identified five themes related to overall feasibility, Table 3.

Themes supporting quantitative findings:

1. Interest/enjoyment.
2. New opportunities.
3. Mastery.
4. Lack of time/availability.

Themes refuting quantitative findings:

5. Challenges in delivering CST.

**Table 3:** Key themes emerging from the semi-structured interviews with 11 participant-dyads

Theme	Quote from diary or interview
New opportunities	I think when she said we’ll do it wherever in the week, so, I was pleased about that. [Participant with PRD, CS6, interview]
	“When it was about topics that we were familiar with, you certainly were stimulated and excited to take part, dad, and afterwards you would be tired because you’ve been active for that time.” [Care partner, CS9, interview]
	It took a lot of pressure off us, for, you know, in as much as it gave us a fresh thing to focus on. [Care partner, CS9, interview]
	Sometimes we were enjoying ourselves that much it got to 45 min. [Care partner, CS5, interview]
	Time spent doing something useful. [Care partner, CS15, diary]
	It’s been great and to say it’s, um, it deals with memories and things that I’ve forgotten about. [Participant with PRD, CS12, interview]
	As well as me doing it and [wife], um, family and friends have asked what we’re doing and it’s, an interest, so we’ve, it gives us a chance to discuss and raise awareness. That, that’s a big benefit already, plus the fact that, um, I, I firmly believe exercise is, is the key, both physical and mental, I don’t do either of them enough so, you know. So, anything that comes along to my doorstep is brilliant, that’s another good plus. [Participant with PRD, CS12, interview]
Interest/enjoyment	And there’s so much choice. There’s so much choice looking through the book, you know, it really is, it’s quite fun deciding, “well, we’ll do this” or whatever, you know. [Care partner, CS12, interview]
	I loved the book. I think it’s lovely, I think everybody should have one. [Care partner, CS12, interview]
	It was very attractively presented I thought. The layout, was, was quite, was quite good. […] They’re all kind of like easy to look at and to follow. [Participant with PRD, CS17, interview]
	I must admit I’ve tended to go for things with pictures rather than too many [words]. […] The pictures are so, oh they’re so nice. This one, “look at this.” [Care partner, CS11, interview]
	To recall some of the things we went through together, as children, as teenagers what have you, I enjoyed it. [Participant with PRD, CS9, interview]
	We really enjoyed, you know, we enjoyed playing games together. [Care partner, CS12, interview]
Mastery	Found it easy [Care partner, CS5, diary]
	As he has travelled considerably he was able to talk about this topic very well. [Care partner, CS1, diary]
	He seemed to enjoy the whole session more today. [Care partner, CS17, diary]
	Some of the questions […] did require more thought. [Participant with PRD, CS17, interview]
	Some of my childhood and things that had gone past, you forgotten all of that you’re only assisted, until we sat down in here and we went through the sort of headlines and it was fantastic to thread those paths again and then I can compare with other people’s experiences. [Participant with PRD, CS9, interview]
Lack of time/availability	3 times a week that was enough, well for us it was enough because we had other things going on in our lives. [Care partner, CS9, interview]
	I think our only barriers were if you weren’t well or I’ve been busy. [Care partner, CS9, interview]
	Well time was the barrier and that I don’t necessary want to do more, one, I think we are more joined at the hip than we would want to be anyway so actually more intensive therapy is the last thing I want whereas when we’re in a group the dynamics is very different. [Care partner, CS5, interview]
	I think, the ones [activities] that we got into there wasn’t enough time. [Participant with PRD, CS5, interview]
	Timing was wrong for me as I was in the middle of something. [Care partner, CS4, diary]
	Feeling under pressure due to time constraints. [Care partner, CS3, diary]
Challenges in delivering CST-PD (discourse)	I probably didn’t like some of the wording in the, and I can’t even pick anything specific. I struggled a bit with some of them and I’d look and think hmmm, it felt a bit stilted with the questions. [Care partner, CS6, interview]
	I wanted to get a discussion going and it didn’t come out as a discussion, it was more questioning and [him] answering. [Care partner, CS17, interview]
	Have we done it right? I felt sometimes that maybe I was the problem. Maybe I wasn’t approaching this right and I wasn’t getting the best out of [him]. [Care partner, CS4, interview]
	You’d come to the question and you’d say, “Well, I’ve already said that”, so what else can I say? [Participant with PRD, CS5, interview]
	I’m thinking ‘if this is supposed to last 30 min there was no way it could have lasted that long’ […] [the topic] was quite, erm, quite a short one and I’m thinking well how can you flesh that out? [Care partner, CS6, interview]
	I struggled on this topic as I was not familiar with all the seven wonders. […] I was not able to give him the support he needed. [Care partner, CS6, diary]
	Trying to make it last 30 min! [Care partner, CS3, diary]
	Didn’t have much to discuss because of our lack of knowledge. [Care partner, CS5, diary]
	Thought he was back at school, didn’t like this. [Care partner, CS8, diary]
	Just thought it was boring. [Care partner, CS8, diary]
Challenges in delivering CST-PD (managing disease symptoms)	Sometimes I said, I was fine to do it but I know he wouldn’t because he was tired […] so it is difficult, more than you think. At the beginning I thought “3 sessions that’s going to be easy” towards the end it was getting to two. But then I thought we’d rather have two good sessions rather than fitting it in for the sake of it. [Care partner, CS14, interview]
	Took 35/40 min, needs condensing, [he] became restless. [Care partner, CS7, diary]
	We had to stop because [name] was “out of it” - not really well, hence only one session this week. [Care partner, CS11, diary]
	[name]’s apparent early loss of interest. [Care partner, CS10, diary]
	It highlighted how [name] needs a strong regular interest in a topic to be truly engaged. [Care partner, CS16, diary]
	Needed prompting at times, didn’t take the lead role! [Care partner, CS14, diary]
	[name] took more interest in the subject today and it was less like “pulling teeth”. [Care partner, CS17, diary]
	There were a lot of components to this question, he got tired after the first page. [Care partner, CS2, interview]
	It was quite difficult for the person I look after to understand the task. [Care partner, CS3, diary]
	I think it did become tired, didn’t it, you know, um, there were occasions where we said, well, I think we’ve done enough for today, I think let’s finish it off tomorrow. [Participant with PRD, CS17, interview]
	C: Perhaps you get a bit tired. P: Weary. C: Well, it wasn’t […] the project’s fault, it was just us not getting the balance quite right. [Participant with PRD and Care partner, CS5, interview]

## Discussion

This is the first report of a psychosocial therapy specifically adapted for people with PDD, DLB or PD-MCI and their care partners. The intervention, designed to be pragmatic, flexible and low cost, involved collaboration and cooperation between the person with PRD and their care partner. In sum, we found that undertaking a RCT of the intervention was feasible and that the intervention itself was acceptable and well tolerated by both members of the participant-dyad, although certain modifications will be needed in a future trial.

For the components for the trial which we classified as ‘modify’ on the ACCEPT checklist, possible modifications for a future trial would include: (1) the trial design should include more flexibility in timing of visits since arranging home visits in older, potentially frail participants often incurs delays; (2) the recruitment target, while achieved, was ambitious, and a rate of one dyad per site per 6 weeks may be more realistic; (3) to account for variations in delivery of the intervention, as well as certain challenges faced by care partners in the role of therapists, inclusion of lay therapists or volunteer therapists should be considered; this notion was raised at a recent post-study Patient and Public Voice (PPV) event and met with general approval from the group; (4) strategies to increase the number of sessions per week from two to three could include more engaging, digitised presentations of the therapy sessions with dashboards to record progress and number of sessions achieved; and (5) certain components involving communication skills or a reliance on motor skills could be modified to obviate these aspects since this proved a challenge for certain participants with communication or physical impairments.

Recruitment was challenging because of the overall frailty of the study population; however, we achieved our threshold for feasibility, eligibility and retention. This was a key finding, particularly since our pre-randomisation refusal of the invitation to participate in the study was high (50%). It is likely that the relatively high degree of physical frailty of the study population, as well as extent of care partner burden, may have contributed to this high rate of refusal. Nonetheless, our recruitment target was reached since many potential participants, as well as referring clinicians recognised the importance of such an intervention, and few opportunities for non-medication-based interventions for people with PD-MCI/PDD/DLB exist. Additionally, most dyads who were initially allocated to the TAU arm expressed interest to receive the intervention following their TAU participation but the decline in their health made them rethink their decision. This highlights the challenges of conducting research in this ever-changing and frail population. Randomisation and blinding were both feasible, although slight imbalance in group characteristics may have been due to the relatively modest number of participant-dyads randomised or the lack of stratification by site. The majority of exploratory assessments were feasible.

The majority of the study design procedures were also feasible. The process evaluation revealed that participant-dyads in the CST-PD arm remained in the trial longer than expected due to difficulty in scheduling the therapy training session. A future study could offer a more flexible therapy training option using online or teleconferencing resources.

Regarding intervention fidelity, our quantitative data indicated that care partners had acquired sufficient knowledge to be able to deliver the therapy; however, our qualitative findings suggested some care partners experienced difficulty personalising and adapting the discussion cues provided in the manual. Our findings regarding enactment indicated that additional strategies are needed to boost adherence. One approach to address both of these aspects would be to include a trained, lay therapist to deliver CST-PD. The lay therapist, perhaps a community-based trained volunteer, could visit the participant-dyad once a week to host an additional session. Observing the lay therapist delivering the therapy effectively may increase therapy-delivery efficacy in the care partner through vicarious experience.

The qualitative findings suggested credibility of CST-PD and possible mechanisms by which this type of intervention may be effective: engendering a sense of mastery through implicit cognitive stimulation that builds on retained abilities and social stimulation with the care partner. For example:

“Some of my childhood and things that had gone past, you forgotten all of that you’re only assisted, until we sat down in here and we went through the sort of headlines and it was fantastic to thread those paths again and then I can compare with other people’s experiences. [Participant with PRD, CS9].

This statement incorporates several potential theoretical mechanisms, both direct and indirect, which are consistent with the multi-faceted nature of the intervention. For example, from the perspective of ‘pure CST’, change may come about through indirect improvements in cognition resulting from being more relaxed, improved self-esteem due to partner encouragement, greater concentration and memory abilities [32, 33]. From the perspective of rehabilitation, change may result from activation of unaffected neural pathways as well as optimisation of remaining abilities, such as in the mechanisms of cognitive rehabilitation therapy applied to dementia [34]. Finally, an important component here is the dyadic aspect and the work undertaken with the spouse, and the person-centred approach resulting from the tailoring of the intervention. The mechanisms underlying dyadic therapies related to improving communication, interpersonal relationships and even intimacy [35, 36]. Quality of life, as a multi-dimensional outcome of such interventions, will be affected by all these factors: improved cognition and functional ability, reversal of apathy, and improved communication and relationships with care partners [33, 37].

Our study had some limitations that need to be considered. Firstly, since CST-PD was delivered at home by a care partner, we were unable to account in detail for the quality and content of each therapy session, and this is one of the aspects of the intervention that will require modification. Secondly, the imbalance in the number of males compared to female participants may have influenced the reports of acceptability and tolerability of the intervention. This point is important and men with PD are more often studied than women, even out of proportion to the actual prevalence ratio of 2:1; our male: female ratio was still higher at 1:3.75. If studying outcomes such as cognition (e.g., verbal fluency), in which gender differences have been reported [38], such imbalances need to be considered. In addition, there may be some differences in males as compared to females in how PD symptoms affect their lives socially, and this may affect aspects of the intervention. For example, facial masking in women may be contrary to social norms for expressivity in women but not in men, resulting in problems with social relationships for people with PD [39]; however, since our intervention was highly tailored to the individual needs of the participants, such issues and other gender-related aspects are not likely to be significant. Finally, we have found no evidence in the literature of a gender difference in tolerability or acceptability of psychosocial therapies. Stratified randomisation in a future effectiveness trial may preclude such imbalances in baseline characteristics.

In conclusion, our iterative approach to developing and piloting an adapted form of CST is consistent with guidance and represents an important step in the emerging field of psychosocial therapies for this population.

## Electronic supplementary material

Below is the link to the electronic supplementary material.

Supplementary material 1 (DOCX 21 KB)

Supplementary material 2 (DOCX 39 KB)

Supplementary material 3 (DOCX 20 KB)
