# Supplementary material for: Parkinson’s-adapted cognitive stimulation therapy: feasibility and acceptability in Lewy body spectrum disorders
Source: J Neurol. 2019 Jun 4;266(7):1756–70. doi: 10.1007/s00415-019-09329-6 (PMC6586694; doi:10.1007/s00415-019-09329-6)
Supplement: Supplementary file 1 — Supplementary material 1 (DOCX 21 KB) [file 415_2019_9329_MOESM1_ESM.docx]

Submission to Journal of Neurology

**Parkinson’s-adapted Cognitive Stimulation Therapy:**

**Feasibility and acceptability in Lewy body spectrum disorders**

Sheree A. McCormick, PhD^1^; Sabina Vatter, MA^1^; Lesley-Anne Carter, PhD^2^; Sarah J. Smith, PhD^3^; Vasiliki Orgeta, PhD^4^; Ellen Poliakoff, PhD^1^; Monty A. Silverdale, MD, PhD^5^; Jason Raw, MD^6^; David J. Ahearn, MD^7^; Christine Taylor, MD^8^; Joanne Rodda, MD^9^; Tarek Abdel-Ghany, MD^10^; Iracema Leroi^1,11*^.

^1^ Division of Neuroscience and Experimental Psychology, University of Manchester, UK;

^2^ Division of Population Health, Health Services Research & Primary Care, University of Manchester, UK

^3^ School of Health and Community Studies, Leeds Beckett University, UK

^4^ Division of Psychiatry, University College London, UK

^5^ Salford Royal NHS Foundation Trust, UK

^6^ Pennine Acute Hospitals NHS Trust, UK

^7^ Manchester University NHS Foundation Trust, UK

^8^ Derbyshire Healthcare NHS Foundation Trust, UK

^9^ North East London NHS Foundation Trust, UK

^10^ North West Boroughs Healthcare NHS Foundation Trust, UK

^11^ Greater Manchester Mental Health NHS Foundation Trust, UK

***Corresponding author:**

Iracema Leroi

Division of Neuroscience & Experimental Psychology,

University of Manchester,

Jean McFarlane Building,

Oxford Road, Manchester

M13 9PL. U.K.

Tel: +44 (0) 161 3067492

Email: [iracema.leroi@manchester.ac.uk](mailto:iracema.leroi@manchester.ac.uk)

**Supplementary Table 1 Themes and topics from the CST-PD manual**

| **CST-PD session theme** | **Specific topics** | |
| --- | --- | --- |
| **1. Personal life** | 1.1 Childhood  1.2 My family  1.3 Relationships | 1.4 Education & Occupation  1.5 Wedding traditions |
| **2. Food** | 2.1 Breakfast  2.2 World cuisine  2.3 Ingredients | 2.4 Then and now  2.5 Staying healthy |
| **3. Hobbies and leisure** | 3.1 My perfect day  3.2 Parks  3.3 Pets  3.4 Water sports  3.5 Ball games  3.6 Winter sports | 3.7 Flowers and trees  3.8 Vegetables and herbs  3.9 In and around the garden  3.10 Libraries and reading  3.11 Space and planets |
| **4. Art** | 4.1 History of art  4.2 Lowry & Turner  4.3 19^th^ century  4.4 Pop art | 4.5 Art from the Islamic world  4.6 Architecture  4.7 Drawing cartoons  4.8 Painting water |
| **5. Media and entertainment** | 5.1 Art inspired by music  5.2 Musical instruments  5.3 Music genres  5.4 Live performances | 5.5 Current affairs: Magazines  5.6 Current affairs: Newspapers  5.7 Current affairs: Reporters  5.8 Technology |
| **6. Nature** | 6.1 Patterns, shapes and colours  6.2 Cloud formations  6.3 Weather conditions | 6.4 Water  6.5 Water and people  6.6 Animal kingdom |
| **7. Seasons** | 7.1 Autumn  7.2 Winter | 7.3 Spring  7.4 Summer |
| **8. Travel and culture** | 8.1 Continents  8.2 UNESCO sites in Europe  8.3 Flags  8.4 Seven Human Wonders  8.5 Public celebrations | 8.6 World celebrations  8.7 Chinese New Year  8.8 Blackpool: Illuminations  8.9 Blackpool: Performances  8.10 Blackpool: The holiday destination |
| **9. Games** | 9.1 Old Wives’ tales  9.2 Being creative  9.3 Tic tac toe  9.4 Match the pairs | 9.5 Decode the sentence  9.6 Proverbs  9.7 Quiz board game  9.8 Colouring and doodling |
