# Supplementary material for: Parkinson’s-adapted cognitive stimulation therapy: feasibility and acceptability in Lewy body spectrum disorders
Source: J Neurol. 2019 Jun 4;266(7):1756–70. doi: 10.1007/s00415-019-09329-6 (PMC6586694; doi:10.1007/s00415-019-09329-6)
Supplement: Supplementary file 2 — Supplementary material 2 (DOCX 39 KB) [file 415_2019_9329_MOESM2_ESM.docx]

Submission to Journal of Neurology

**Parkinson’s-adapted Cognitive Stimulation Therapy:**

**Feasibility and acceptability in Lewy body spectrum disorders**

Sheree A. McCormick, PhD^1^; Sabina Vatter, MA^1^; Lesley-Anne Carter, PhD^2^; Sarah J. Smith, PhD^3^; Vasiliki Orgeta, PhD^4^; Ellen Poliakoff, PhD^1^; Monty A. Silverdale, MD, PhD^5^; Jason Raw, MD^6^; David J. Ahearn, MD^7^; Christine Taylor, MD^8^; Joanne Rodda, MD^9^; Tarek Abdel-Ghany, MD^10^; Iracema Leroi^1,11*^.

^1^ Division of Neuroscience and Experimental Psychology, University of Manchester, UK;

^2^ Division of Population Health, Health Services Research & Primary Care, University of Manchester, UK

^3^ School of Health and Community Studies, Leeds Beckett University, UK

^4^ Division of Psychiatry, University College London, UK

^5^ Salford Royal NHS Foundation Trust, UK

^6^ Pennine Acute Hospitals NHS Trust, UK

^7^ Manchester University NHS Foundation Trust, UK

^8^ Derbyshire Healthcare NHS Foundation Trust, UK

^9^ North East London NHS Foundation Trust, UK

^10^ North West Boroughs Healthcare NHS Foundation Trust, UK

^11^ Greater Manchester Mental Health NHS Foundation Trust, UK

***Corresponding author:**

Iracema Leroi

Division of Neuroscience & Experimental Psychology,

University of Manchester,

Jean McFarlane Building,

Oxford Road, Manchester

M13 9PL. U.K.

Tel: +44 (0) 161 3067492

Email: [iracema.leroi@manchester.ac.uk](mailto:iracema.leroi@manchester.ac.uk)

**Supplementary Table 2 Outcome measure descriptions for people with Parkinson’s-related dementia and care partners**

|  |  |  | **Respondent** | |
| --- | --- | --- | --- | --- |
| **Outcome domain** | **Specific measurement tool** | **Description of the tool** | **Person with PRD** | **Care partners** |
| Cognition | **The Addenbrooke’s Cognitive Examination** (ACE-III) ^a^ [1] | Global cognition (total score) and cognitive sub-domains of memory, attention, verbal fluency, language and visuospatial function. | ✓ |  |
|  | **The Dementia Cognitive Fluctuation Scale** (DCF) ^b^ [2] | Fluctuations in person with PRD cognition reported by the care partner. | ✓  (proxy) |  |
| Functional ability | **The Pill questionnaire** ^b^ [3] | The ability to undertake a specific activity of daily living (i.e. medication intake). | ✓ |  |
| Quality of life | **The Parkinson’s Disease Questionnaire-39** (PDQ-39) ^b^ [4] | Parkinson’s-specific quality of life. | ✓ |  |
|  | **The EuroQoL-5D** (EQ5D) ^a^ [5] | Health-related quality of life. | ✓ | ✓ |
| Neuropsychiatric symptoms (NPS) | **The Neuropsychiatric Inventory** (NPI) ^b^ [6] | Presence and magnitude of ‘clinically significant’ (frequency x severity ≥4) NPS sub-domains reported by the care partner. | ✓  (proxy) |  |
|  | **The Hospital Anxiety and Depression Scale** (HADS) ^b^ [7] | Self-rated anxiety and depression. | ✓ | ✓ |
|  | **The Lille Apathy Rating Scale** (LARS) ^b^ [8] | Self-rated apathy. | ✓ |  |
| Health | **The Short Form-12 Health Survey** (SF-12) ^a^ [9] | General physical and mental health. |  | ✓ |
| Relationship quality | **The Relationship Satisfaction Scale** (RSS) ^a^ [10] | Satisfaction with the dyadic relationship. | ✓ | ✓ |
|  | **The Dyadic Relationship Scale** (DRS) ^a,b^ [11] | Positive dyadic interaction and negative strain. |  | ✓ |
|  | **The Family Caregiving Role Scale** (FCR) ^a,b^ [12] | Specific feelings associated to care provision. |  | ✓ |
| Burden | **The Zarit Burden Interview** (ZBI) ^b 13^ [13] | Burden related to care provision. |  | ✓ |
|  | **The Relatives’ Stress Scale** (Rel.SS) ^b^ [14] | Stress related to care provision. |  | ✓ |
| Resilience | **The Brief Resilience Scale** (BRS) ^a^ [15] | The ability to bounce back in stressful situations. | ✓ | ✓ |
| Empathy | **The Interpersonal Reactivity Index** (IRI) ^a^ [16] | Empathic tendencies and perspective taking. | ✓ |  |

^a^ Higher scores better, ^b^ Higher scores worse

1. Hsieh S, Schubert S, Hoon C, et al (2013) Validation of the Addenbrooke’s Cognitive Examination III in Frontotemporal Dementia and Alzheimer’s Disease. Dement Geriatr Cogn Disord 36:242–250. https://doi.org/10.1159/000351671

2. Lee DR, McKeith I, Mosimann U, et al (2014) The Dementia Cognitive Fluctuation Scale, a New Psychometric Test for Clinicians to Identify Cognitive Fluctuations in People with Dementia. Am J Geriatr Psychiatry 22:926–935

3. Reginold W, Armstrong MJ, Duff-Canning S, et al (2012) The pill questionnaire in a nondemented Parkinson’s disease population. Mov Disord 27:1308–1311. https://doi.org/10.1002/mds.25124

4. Jenkinson C, Fitzpatrick R, Peto V, Greenhall R (1997) The Parkinson ’ s Disease Questionnaire ( PDQ-39 ): development and validation of a Parkinson ’ s disease summary index score. Age Ageing 26:353–357

5. EuroQol Group (1990) EuroQol--a new facility for the measurement of health-related quality of life. Health Policy 16:199–208. https://doi.org/10.1016/0168-8510(90)90421-9

6. Cummings, J. L., Mega, M., Gray, K., Rosenberg-Thompson, S., Carusi, D. A., & Gornbein J (1994). (1994) The neuropsychiatric inventory: Comprehensive assessment of psychopathology in dementia. Neurology 44:2308–2314

7. Zigmond AS, Snaith PR (1983) The hospital anxiety and depression scale. 67:361–370. https://doi.org/10.1111/j.1600-0447.1983.tb09716.x

8. Sockeel P, Dujardin K, Devos D, et al (2006) The Lille apathy rating scale (LARS), a new instrument for detecting and quantifying apathy: validation in Parkinson’s disease. J Neurol Neurosurg Psychiatry 77:579–84. https://doi.org/10.1136/jnnp.2005.075929

9. Ware J, Kosinski M, Keller SD (1996) A 12-Item SHort Health Survey: construction of scales and preliminary tests of reliability and validity. Med Care 34:220–33. https://doi.org/10.1097/00005650-199603000-00003

10. David B (1983) Ten days to self-esteem. Quill William Morrow, New York

11. Sebern MD, Whitlatch CJ (2007) Dyadic relationship scale: a measure of the impact of the provision and receipt of family care. Gerontologist 47:741–51. https://doi.org/10.1093/geront/47.6.741

12. Schofield HL, Murphy B, Herrman HE, et al (1997) Family caregiving: measurement of emotional well-being and various aspects of the caregiving role. Psychol Med 27:647–57. https://doi.org/10.1017/S0033291797004820

13. Zarit SH, Reever KE, Bach-Peterson J (1980) Relatives of the Impaired Elderly: Correlates of Feelings of Burden. Gerontologist 20:649–655. https://doi.org/10.1093/geront/20.6.649

14. Greene JG, Smith R, Gardiner M, Timbury GC (1982) Measuring behavioural disturbance of elderly demented patients in the community and its effects on relatives: a factor analytic study. Age Ageing 11:121–126. https://doi.org/10.1093/ageing/11.2.121

15. Smith BW, Dalen J, Wiggins K, et al (2008) The brief resilience scale: Assessing the ability to bounce back. Int J Behav Med 15:194–200. https://doi.org/10.1080/10705500802222972

16. Davis MH (1983) A Multidimensional Approach to Individual Differences in Empathy. J Pers Soc Psychol 44:113–126. https://doi.org/10.1037/0022-3514.44.1.113
