# Supplementary material for: Parkinson’s-adapted cognitive stimulation therapy: feasibility and acceptability in Lewy body spectrum disorders
Source: J Neurol. 2019 Jun 4;266(7):1756–70. doi: 10.1007/s00415-019-09329-6 (PMC6586694; doi:10.1007/s00415-019-09329-6)
Supplement: Supplementary file 3 — Supplementary material 3 (DOCX 20 KB) [file 415_2019_9329_MOESM3_ESM.docx]

Submission to Journal of Neurology

**Parkinson’s-adapted Cognitive Stimulation Therapy:**

**Feasibility and acceptability in Lewy body spectrum disorders**

Sheree A. McCormick, PhD^1^; Sabina Vatter, MA^1^; Lesley-Anne Carter, PhD^2^; Sarah J. Smith, PhD^3^; Vasiliki Orgeta, PhD^4^; Ellen Poliakoff, PhD^1^; Monty A. Silverdale, MD, PhD^5^; Jason Raw, MD^6^; David J. Ahearn, MD^7^; Christine Taylor, MD^8^; Joanne Rodda, MD^9^; Tarek Abdel-Ghany, MD^10^; Iracema Leroi^1,11*^.

^1^ Division of Neuroscience and Experimental Psychology, University of Manchester, UK;

^2^ Division of Population Health, Health Services Research & Primary Care, University of Manchester, UK

^3^ School of Health and Community Studies, Leeds Beckett University, UK

^4^ Division of Psychiatry, University College London, UK

^5^ Salford Royal NHS Foundation Trust, UK

^6^ Pennine Acute Hospitals NHS Trust, UK

^7^ Manchester University NHS Foundation Trust, UK

^8^ Derbyshire Healthcare NHS Foundation Trust, UK

^9^ North East London NHS Foundation Trust, UK

^10^ North West Boroughs Healthcare NHS Foundation Trust, UK

^11^ Greater Manchester Mental Health NHS Foundation Trust, UK

***Corresponding author:**

Iracema Leroi

Division of Neuroscience & Experimental Psychology,

University of Manchester,

Jean McFarlane Building,

Oxford Road, Manchester

M13 9PL. U.K.

Tel: +44 (0) 161 3067492

Email: [iracema.leroi@manchester.ac.uk](mailto:iracema.leroi@manchester.ac.uk)

**Supplementary Table 3 Companion’s CST-PD diary excerpt**

Please select the most appropriate response to the statements below:

| **During the session….** | **Strongly Disagree** | **Disagree** | **Neutral** | **Agree** | **Strong Agree** |
| --- | --- | --- | --- | --- | --- |
| your partner was interested. | 1 | 2 | 3 | 4 | 5 |
| your partner was motivated. | 1 | 2 | 3 | 4 | 5 |
| your partner displayed emotional responses.* | 1 | 2 | 3 | 4 | 5 |
| your partner took the initiative (at times). | 1 | 2 | 3 | 4 | 5 |
| your partner gained a sense of achievement. | 1 | 2 | 3 | 4 | 5 |

*(**anger, disgust, fear, happiness, surprise, sadness)
